# Properties of vertebrate predator–prey networks in the high Arctic

**DOI:** 10.1002/ece3.11470

**Published:** 2024-05-30

**Authors:** Muzit Abrham, Karin Norén, Jordi Bartolomé Filella, Anders Angerbjörn, Nicolas Lecomte, Patrícia Pečnerová, Susana Freire, Fredrik Dalerum

**Affiliations:** ^1^ Department of Zoology Stockholm University Stockholm Sweden; ^2^ Department of Animal and Food Science Autonomous University of Barcelona Barcelona Spain; ^3^ Department of Biology University of Moncton Moncton New Brunswick Canada; ^4^ Department of Biology University of Copenhagen Copenhagen Denmark; ^5^ Biodiversity Research Institute CSIC‐Univ. Oviedo‐Princ Mieres Spain; ^6^ Biodiversity Research Institute Mieres Spain; ^7^ Mammal Research Institute, Department of Zoology and Entomology University of Pretoria Pretoria South Africa

**Keywords:** Ellesmere Island, Greenland, modularity, nestedness, predation, trophic network

## Abstract

Predation is an important ecological process that can significantly impact the maintenance of ecosystem services. In arctic environments, the relative ecological importance of predation is thought to be increasing due to climate change, partly because of increased productivity with rising temperatures. Therefore, understanding predator–prey interactions in arctic ecosystems is vital for the sustainable management of these northern regions. Network theory provides a framework for quantifying the structures of ecological interactions. In this study, we use dietary observations on mammalian and avian predators in a high arctic region, including isolated peninsulas on Ellesmere Island and north Greenland, to construct bipartite trophic networks. We quantify the complexity, specialization, and nested as well as modular structures of these networks and also determine if these properties varied among the peninsulas. Mammal prey remains were the dominant diet item for all predators, but there was spatial variation in diet composition among peninsulas. The predator–prey networks were less complex, had more specialized interactions, and were more nested and more modular than random expectations. However, the networks displayed only moderate levels of modularity. Predator species had less specialized interactions with prey than prey had with predators. All network properties differed among the peninsulas, which highlights that ecosystems often show complex responses to environmental characteristics. We suggest that gaining knowledge about spatial variation in the characteristics of predator–prey interactions can enhance our ability to manage ecosystems exposed to environmental perturbations, particularly in high arctic environments subject to rapid environmental change.

## Graphical abstract

Predation is an important ecological process, particularly in arctic environments, which undergo rapid environmental change. Vertebrate trophic networks from a fragmented terrestrial ecosystem on northern Ellesmere Island and north Greenland were less complex, had more specialized interactions, and were more nested and more modular than random expectations. Networks also varied among several isolated peninsulas, which highlights the often‐complex ecological responses to environmental characteristics.

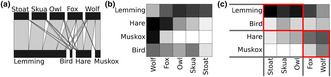

## 1 INTRODUCTION

Predation is an important ecological process that influences many ecosystem functions (Miller et al., 2001; Ritchie & Johnson, 2009; Terborgh et al., 1999). Predation can influence ecosystem properties both directly by prey being killed and indirectly by altering prey behavior, morphology, and physiology through responses to predation risk (Creel & Christianson, 2008; Estes et al., 2011; Lima, 1998; Taylor, 1984). The combined direct and indirect effects of predators on prey species can have cascading effects on ecosystems (Ray et al., 2005; Schmitz & Suttle, 2001) and profoundly alter the environments in which predator–prey interactions take place (Ripple et al., 2014).

Predation has an important role in regulating terrestrial arctic ecosystems (Ims et al., 2013). The relative importance of predation as a regulatory force in arctic ecosystems is also expected to increase with a warmer climate due to enhanced primary productivity (Legagneux et al., 2014). Arctic regions are characterized by a harsh environment with relatively simple ecosystems, whose simplicity is primarily caused by low productivity (Ims et al., 2013; Juhasz et al., 2020). However, cold regions such as the Arctic are experiencing a more rapid increase in temperature than other regions (Comiso & Hall, 2014; Hamilton et al., 2015; Kausrud et al., 2008; Serreze & Barry, 2011). Consequently, climate change is likely to have significant impacts on the dynamics and structure of arctic ecosystems by altering the characteristics of predation processes (Elmhagen et al., 2015; Hamilton et al., 2017; Nolet et al., 2013; Stirling & Derocher, 2012; Zimova et al., 2016). Knowledge about the structure of predator–prey interactions in arctic ecosystems is therefore crucial for our ability to conserve arctic biodiversity in the face of ongoing and future climate change (Schmidt et al., 2017; Van der Putten et al., 2004; Woodward et al., 2010).

One prominent characteristic of terrestrial northern ecosystems is strong temporal fluctuations in the population sizes of many potential prey species, for example, microtone rodents (Arvicolinae, Krebs et al., 2002) and snowshoe hares (*Lepus americanus*, Krebs et al., 2001), as well as large ungulates such as reindeer (*Rangifer tarandus*) and muskoxen (*Ovibus moschatus*, Forchhammer et al., 2002). However, such temporal dynamics may not always be synchronized across regional (Angerbjörn et al., 2001) or local scales (Gruyer et al., 2008; Vigués et al., 2022). Such asynchrony can influence both population characteristics (Engen et al., 2002; Gaillard et al., 2000; Heino et al., 1997) and community dynamics (Boutin, 1995) and cause spatial variation in predator–prey interactions (e.g., Dalerum et al., 2018).

Ecological interactions can be depicted as networks, a type of mathematical graph in which the interacting organisms are represented as nodes, or vertices, and their interactions as links, or edges (Dale, 2017; Proulx et al., 2005). The structures of ecological interactions identified from such networks can reveal important information about ecosystem properties and can be analyzed quantitatively (Delmas et al., 2019; Dale & Fortin, 2021). For instance, both the complexity as well as the structures of networks depicting ecological interactions have been linked to ecological resilience, although for different reasons (Thébault & Fontaine, 2010). For predator–prey interactions, networks are usually depicted using nodes at two distinct levels reflecting predators and prey, so‐called bipartite networks (Miranda et al., 2013). Since analysis of these types of bipartite networks typically focuses on identifying and quantifying the structures of ecological interactions, it differs from food web approaches, which usually depict nodes at only one level with relatively fixed structures of links among them. Studies using this latter approach typically focus on quantifying relative link strengths as a measure of energy or nutrient transfer (Lindeman, 1942).

In this study, we use bipartite networks to quantify the properties of trophic interactions between mammalian and avian predators and their prey in land areas surrounding the Nares Strait. This region includes geographically separated peninsulas on Ellesmere Island, Canada, and north Greenland. It represents one of the northernmost terrestrial regions on Earth. Many different metrics exist to quantify the properties of bipartite networks (Dale & Fortin, 2021). We have chosen to focus on network complexity, the level of specialization in ecological interactions, the level of nestedness in the interaction structures, and the level of modularity in the interaction structures. We selected these metrics not only due to their ecological relevance but also because they are complementary in describing the properties of the predator–prey interactions we aim to describe (Dalerum et al., 2016; Delmas et al., 2019). Complexity, nestedness, and modularity were only quantified at the full network level, whereas interaction specialization was quantified at all three levels, that is, at the network‐, trophic (i.e. separately for predators and prey) and node levels. Network complexity describes realized network size in relation to some theoretical maximal size (Dunne et al., 2002), and interaction specialization, in the implementation we have used, describes the relative selectivity among the predators in what prey they feed on and the uniqueness of prey in terms of what predators feed on them (Blüthgen et al., 2006). Nestedness and modularity are two interaction structures that are ecologically relevant (Thébault & Fontaine, 2010). In a nested structure, prey used by specialist predators are subsets of prey used by more generalist predators (Ulrich et al., 2009). In a modular, or compartmentalized, interaction structure, predators and prey are instead divided into sub‐communities, where there are more frequent and stronger interactions within each sub‐community than between them (May, 1973).

This high arctic region harbors few species and has low productivity (Ims et al., 2013). Low species diversity tends to result in networks of limited complexity (Page, 2010), and low productivity tends to favor generalist predation strategies (Poisot et al., 2011). Despite the tendency for antagonistic interactions to form modular interaction structures (Bascompte et al., 2003), a high proportion of generalist predators are more likely to cause nested structures of predator–prey interactions. Furthermore, predators are under strong selection pressure to optimize predation strategies, whereas prey are under strong selection pressures to avoid predation, irrespective of the predator (Abrams, 2000). Therefore, it can be expected that predators are more specific in their use of prey than prey are in the predators that are preying on them. Finally, terrestrial areas in this high arctic region are spatially fragmented, and we have previously noted that this spatial fragmentation may have led to asynchronous population dynamics among potential prey species (Dalerum et al., 2017), and subsequent spatial variation in predator–prey interactions (Dalerum et al., 2018). We therefore evaluate the following specific hypotheses: (i) limited predator and prey diversity will result in predator–prey networks with low complexity; (ii) the observed predator–prey networks will reflect low interaction specialization; (iii) however, predators will exhibit a higher level of specialization in their interaction with prey than prey in their interactions with predators; (iv) a high proportion of generalist predators will instead generate nested interaction structures; and (v) asynchronous population dynamics among prey on the different peninsulas will generate differences in the network properties among peninsulas.

## 2 MATERIALS AND METHODS

### 2.1 Study area

**FIGURE 1:**
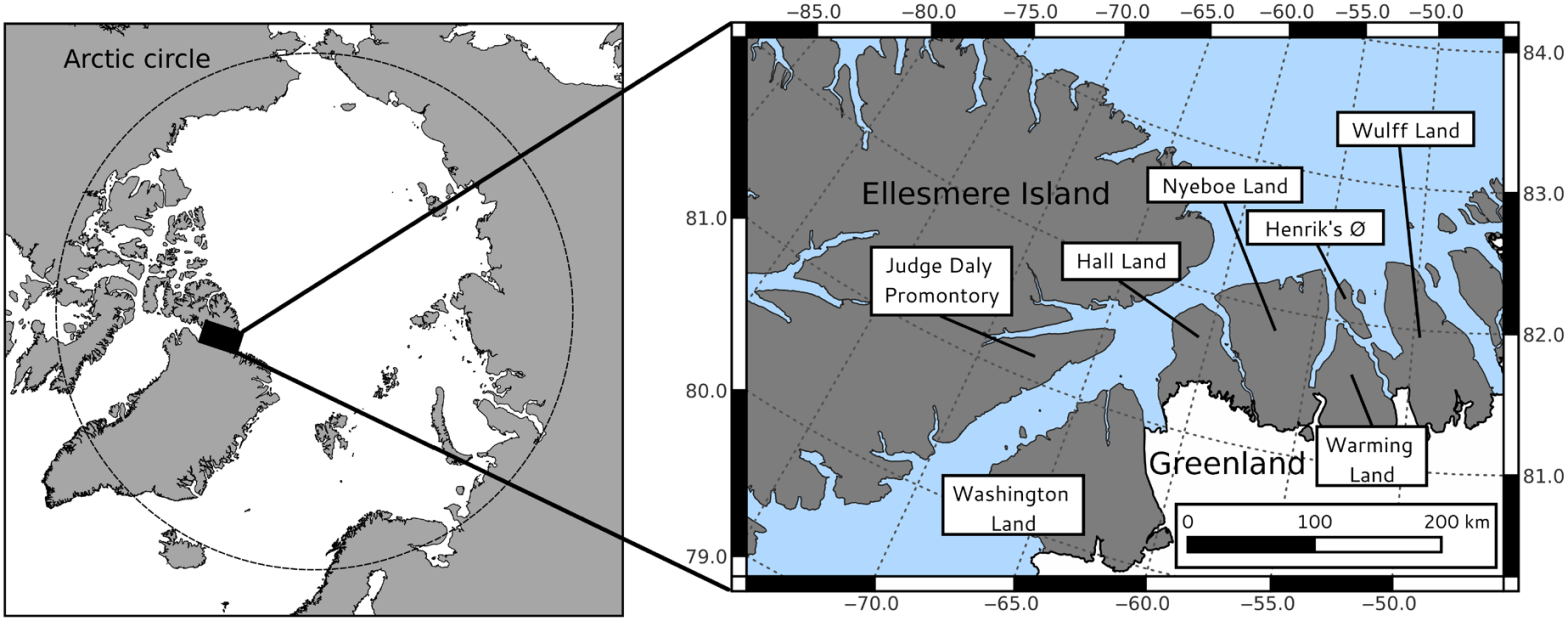
Location of the study sites in high arctic Canada and Greenland visited during expeditions 2015 and 2019. Data from Judge Daly Promontory came from the expedition 2015, data from Washington Land and Hall Land came from both expeditions, and data from Nyeboe Land, Warming Land, Henrik Ø, and Wulff Land only came from the expedition 2019.

Our study is based on samples collected on Judge Daly Promontory on Ellesmere Island, high arctic Canada, and on a series of peninsulas in north Greenland (Figure 1): Washington Land, Hall Land, Nyeboe Land, Warming Land, and Wulff Land. Samples were also collected at Henrik's Ø, a small island between Nyeboe Land and Warming Land. The peninsulas are geographically separated from each other, which causes the terrestrial ecosystem in this region to be fragmented, although they are potentially connected during winter through pack‐ and fjord ice. These land areas emerged about 9500–8000 years ago and are separated by ice shelves, deep fjords, and glaciers (Dick, 2001). Hall Land emerged due to a postglacial rebound (England, 1985), while both Judge Daly promontory and the other areas in Greenland emerged from the deglaciation of the Greenland ice sheet. However, they all contain similar topography, fauna, and flora. The climate is harsh, with a short growing season. The average summer temperature is −1.5°C and the average winter temperature −32°C (Przybylak, 2003). The landscape is dominated by mountains intersected by undulating valleys. Vegetation is sparse, with a mean cover of 10%, and characterized by low‐growth willow (*Salix arctica*), various *Carex* species, and grasses (Dalerum et al., 2017).

On all peninsulas, the terrestrial mammal communities consist of caribou (*Rangifer tarandus*, most likely only occurring on Ellesmere Island and Washington Land), muskox (*Ovibos moschatus*), Arctic hare (*Lepus arcticus*), northern collared lemming (*Dicrostonyx groenlandicus*, hereafter referred to as “lemming”), wolf (*Canis lupus*), Arctic fox (*Vulpes lagopus*), and stoat (*Mustela erminea*). The bird communities include the resident rock ptarmigan (*Lagopus muta*) and common raven (*Corvus corax*), the nomadic snowy owl (*Bubo scandiacus*), as well as a series of seasonal migrants: snow goose (*Chen caerulescens*), brent goose (*Branta bernicla*), king eider (*Somateria spectabilis*), red‐throated diver (*Gavia stellata*), glaucous gull (*Larus hyperboreus*), parasitic skua (*Stercorarius parasiticus*), long‐tailed skua (*S. longicaudus*), Arctic tern (*Sterna paradisaea*), turnstone (*Arenaria interpres*), dunlin (*Calidris alpina*), sanderling (*Calidris alba*), snow bunting (*Plectrophenax nivalis*), lapland longspur (*Calcarius lapponicus*), northern wheatear (*Oenanthe oenanthe*), and gyrfalcon (*Falco rusticolus*) (Dalerum et al., 2017; Rodewald, 2023).

### 2.2 Sample collection

During two icebreaker‐based expeditions, one in 2015 (conducted July–September) and one in 2019 (conducted August–September), we collected feces and regurgitated pellets from all present mammalian and most avian predators occurring in the region: wolf, Arctic fox, stoat, snowy owl, and skuas (*Stercorarius* sp.). Of the avian predators, we did not have samples from gyrfalcons, common ravens or glaucus gulls, even if these species may prey on small vertebrates such as small rodents. However, neither ravens nor gulls primarily function as predators, and gyrfalcons occur at extremely low densities in these northern areas. Hence, we do not regard their omission as substantially influencing the quantification of predator–prey interactions in this region. Three peninsulas were visited in 2015 (Judge Daly Promontory, Washington Land, and Hall Land) and five in 2019 (Washington Land, Hall Land, Nyeboe Land, Warming Land, and Wulff Land). We also visited Henrik's Ø in 2019. Samples were collected by walking the landscape on foot and targeting structures that could be used as dens or nests to maximize our chances of encountering samples. Subsequently, most wolf and Arctic fox feces were collected at dens and carcass sites, and most of the pellets from avian predators were collected from nest sites and conspicuous outcrops. Stoat feces were partly identified inside lemming winter nests (Duchesne et al., 2011). For all species, some of the samples could have come from the same individuals. The total search area varied on the different peninsulas, but thanks to helicopter transport we could cover widely distributed areas even within single peninsulas. We collected all encountered feces and pellets of vertebrate predators, irrespective of how old they appeared to be. Since feces and pellets last for several years in these northern environments, we have no reason to believe that the timing of sample collection on the different peninsulas would have biased our results. Instead, we argue that our results represent an average over several demographic phases of predator and prey populations.

### 2.3 Diet quantification

Collected feces and regurgitated pellets were broken apart, washed in water over a 0.5‐mm mesh, and dried at 60°C for at least 24 h before further examination. The prey remains in each sample were grouped into distinct fractions which only contained a single prey category each. These were then assigned to one of the following categories: muskox, Arctic fox, Arctic hare, collared lemming, unknown mammal, Anseriformes, Galliformes, Charadriiformes, Passeriformes, unknown bird, plants, arthropods, and miscellaneous. Each fraction was identified using bones, hairs, teeth, and feathers (Errington, 1930), either macroscopically or under a microscope, with the aid of reference collections (Dalerum et al., 2018) and available guides (Brom, 1986; Miller & Broughton, 2016; Teerink, 2003). Most mammalian prey remains were identified to the species level (99.99%), while only 52% of avian prey remains were identified to the order level. Plant remains were categorized at the kingdom level, and arthropods were grouped as Arthropoda. Miscellaneous items such as stones, eggshells, and soil were grouped together as a miscellaneous category. Any mammal or avian prey that could not be reliably identified was put into the “unknown mammal” and “unknown bird” categories.

Due to the often‐heavy sample fragmentation, the diet was analyzed in sample units representing the approximate size of a full fecal or pellet unit from each target predator. For each sample, the relative volume of each fraction was visually estimated to the nearest 1% of the total volume of the sample. The relative occurrences of each prey category were subsequently converted into frequencies of whole sample equivalents by taking the integer value of the sum of all volume percentages divided by 100 (Elmhagen et al., 2000). This method provides a diet quantification that maintains the analytical properties of frequencies, that is, integer counts of individual observations, but retains relative abundance variations within samples containing more than one prey item. It will therefore enable quantitative diet assessments from highly fragmented samples, which otherwise are difficult.

### 2.4 Data analysis

We used generalized linear models with a Poisson error structure and log link to evaluate if diet composition differed among the sample sites. We regard the Poisson error structure as appropriate considering that our data had the properties of frequencies, that is, counts of individual observations of each prey category. We ran one model for each predator species. We fitted fully saturated models including diet category, site, and their two‐way interaction as predictor terms. Each model used the frequency of whole scat equivalents in each diet category as the response. Evaluating the interaction terms in these types of models is equivalent to a chi‐square test of independence and provides a powerful framework for analyzing frequency data (Sokal & Rohlf, 1995). For these analyses, all birds were pooled into one dietary category due to the limited sample size of each specific taxonomic group of birds.

We quantified the structure of predator–prey interactions from quantitative bipartite networks (Miranda et al., 2013). The networks were based on matrices consisting of predators as columns, prey categories as rows, and the percentage diet contribution as cell values. For these matrices, we only included four prey categories: muskox, hare, lemmings, and birds. We constructed seven matrices, one containing pooled data from all sites and one for each specific site except for Henrik's Ø, which did not have sufficient samples.

We used weighted connectance as an index of network complexity. This index evaluates the number of observed links in the whole network relative to the total number of theoretically possible links. In its weighted form, it is quantified as the linkage density, that is, the total number of trophic links per species divided by the number of species in the network (Beckerman et al., 2006; Bersier et al., 2002; Dunne et al., 2002). Hence, it is not dependent on network size.

We quantified how specialized the interactions were in the trophic networks at three levels: for the full network, for each trophic level, and for each species. In addition, we quantified the asymmetry in specialization between the two trophic levels. The interaction specialization of prey is, in this context, referring to how “specialized” a prey species is in terms of the predators that prey on it. Hence, it is not a measure of niche utilization, as it is for the predators, but it still provides a useful measurement of the patterns of predation that the different prey experience. We used the H2 index initially proposed by Blüthgen et al. (2006) to quantify the specialization of the full network, and its species‐level equivalent, *d*, to quantify the level of specialization for each predator and prey species in our networks. The H2 index quantifies the extent to which the observed interactions deviate from those that would be expected given the species' marginal totals of the interaction matrix, that is the sum of all interactions for each species, whereas the *d* index estimates how strongly a species deviates from a random sampling of the possible interaction partners. We also quantified the asymmetry in specialization between the two trophic levels using the metric of specialization asymmetry proposed by Blüthgen et al. (2007), which is quantified so that positive numbers indicate higher specialization in the upper level, in our case the predators, and negative numbers indicate higher specialization in the lower level, in our case the prey. We estimated the specialization within each trophic level as the additive inverse of Horn's index of niche overlap (Horn, 1966). All indices of specialization range from 0, representing no specialization in the network, to a maximal value of 1, representing complete specialization (Blüthgen et al., 2006). It is worth noting that H2 and *d* are not indices of niche breadth. Hence, a predator that feeds on a few prey categories but uses categories that are commonly used by other predators may get a lower score than a predator that feeds on many prey categories but uses categories where this predator is the only species feeding on them.

In addition to complexity and specialization, we also evaluated both nested and modular structures for each network. We used the Weighted‐Interaction Nestedness Estimator (WINE) to quantify the nested structure. WINE estimates the nestedness of bipartite networks through the calculation of Manhattan distances considering the weight of the interactions (Galeano et al., 2009). These distances are calculated on a matrix packed by an algorithm that is based on column and row fill, that is, number of cells with nonzero values along each row, weighted by the respective cell values, in this case, dietary proportions (Lin et al., 2018). The nestedness index WINE ranges from zero, which represents a random structure, to a maximal value of one, which represents maximal nestedness. We used the LPAwb+ algorithm proposed by Beckett (2016) to identify modules in our weighted interaction matrices, and quantified the level of modularity using the weighted implementation of Barber's (2007) *Q*, *Q*_w_ (Dormann & Strauss, 2014). The modularity index *Q*_w_ quantifies the likelihood that two interacting nodes are within one module and ranges from zero, indicating that links within modules are not higher than expected by chance, to a maximal value of one, representing all links within modules are higher than expected by chance.

Comparing raw network metrics to those derived from appropriate null model is an important part of extracting useful information from network analyses (Dormann et al., 2017). To evaluate whether the observed values of weighted connectance, network specialization, asymmetry specialization, trophic and species‐level specializations, nestedness, and modularity were caused by differential prey utilization among predators, we compared the observed values to those estimated from 1000 randomized matrices for each observed matrix. Each randomized matrix was constrained to have the same dimensions and column sums as the original matrices, but the percentage contributions to the diet were allowed to freely be redistributed among the prey categories. Hence, the sum of all contributions of dietary categories was maintained at 100% of each predators diet, but in each randomized network, the dietary proportions were allowed to vary freely among all prey categories. Hence, in our randomized networks, each predator would on average utilize equal proportions of all prey categories, which we regard to be an appropriate null model pattern for testing how predation patterns influence the structures of these predator–prey interactions (Gotelli & Graves, 1996).

To provide a heuristic test of geographic variation in the different network indices, we calculated *D*‐values for each index and network as the difference between the observed values and those from all randomized matrices and compared these among sites using a permutation‐based one‐way ANOVA (Manly & Alberto, 2016).

## 3 RESULTS

Diet estimation of mammalian and avian predators was based on a total of 1428 samples: 92 samples from wolves, 657 samples from Arctic foxes, 386 samples from snowy owls, 262 samples from stoats, and 30 samples from skuas (Table 1). Samples were collected from all included predator species at all peninsulas as well as Hendrik's Ø, except for skuas, for which samples were only collected on Judge Daly Promontory, Washington Land, and Hall Land (Table S1). However, sample sizes for all predator species varied both among species and among sites. Although pooling the sample sizes from all sites allowed for the estimation of dietary proportions for most predators and prey categories with high precision, estimating dietary proportions within individual sites had larger margins of error (Figure S1).

**TABLE 1:** Estimated contributions of all prey categories to the diet of wolf, arctic fox, stoat, snowy owl, and skua for samples from all sites pooled.

	Wolf (*N* = 92)	Arctic fox (*N* = 657)	Stoat (*N* = 262)	Snowy owl (*N* = 386)	Skua (*N* = 30)
Mammal	92%	94%	99%	98%	95%
Muskox	31%	1%	0%	0%	0%
Arctic fox	1%	0%	0%	0%	0%
Hare	48%	38%	1%	6%	3%
Lemming	9%	55%	98%	92%	85%
Unk.mammal	3%	0%	0%	0%	7%
Bird	4%	2%	1%	2%	5%
Anseriformes	0%	1%	0%	<1%	0%
Galliformes	2%	1%	0%	1%	0%
Charadriiformes	1%	<1%	0%	<1%	0%
Passeriformes	0%	<1%	1%	<1%	0%
Unk.bird	1%	<1%	0%	1%	5%
Plants	0%	4%	0%	0%	0%
Arthropods	0%	0%	0%	0%	0%
Miscellaneous	4%	0%	0%	0%	0%

### 3.1 Dietary composition

Mammal prey remains were the dominant diet item, contributing to more than 90% of the diets of all predators. Lemmings were the most important prey item for all predators except the wolf, which instead relied mainly on muskoxen and hare (Table 1). However, there were significant variations in the dietary composition among the sites for all predators (wolf: *χ* ^2^ = 66.68, *df* = 18, *p* < .001; Arctic fox: *χ* ^2^ = 104.69, *df* = 18, *p* < .001; stoat: *χ* ^2^ = 33.45, *df* = 18, *p* < .001; snowy owl: *χ* ^2^ = 36.56, *df* = 18, *p* < .001; skua: *χ* ^2^ = 3.36, *df* = 6, *p* < .001). For the wolf, hare had the highest dietary contribution of all prey categories on Judge Daly Promontory (38%), Washington Land (87%), and Nyeboe Land (80%), whereas muskoxen had the highest contribution to the wolf diet on Hall Land (66%) and Warming Land (44%). Lemmings also contributed to the wolf diet on Hall Land (18%), Judge Daly Promontory (16%), and Washington Land (4%). For all other predators, lemmings had the highest dietary contribution on all sites except for the Arctic fox on Washington Land (Table S1), where hare had the highest dietary contribution (73%). Apart from lemmings, stoat diet also included hare on Washington Land (50%), Hall Land (6%), and Wulff Land (20%), and birds on Nyeboe Land (25%) and Henrik's Ø (1%). Snowy owl diet similarly included hare on all sites except Henrik's Ø, and skua diet included hares on Washington Land. For both snowy owl and skua, birds contributed small amounts to the diet on all sites, or, for skua, on all three sites where samples were collected (Table S1).

### 3.2 Network complexity

**FIGURE 2:**
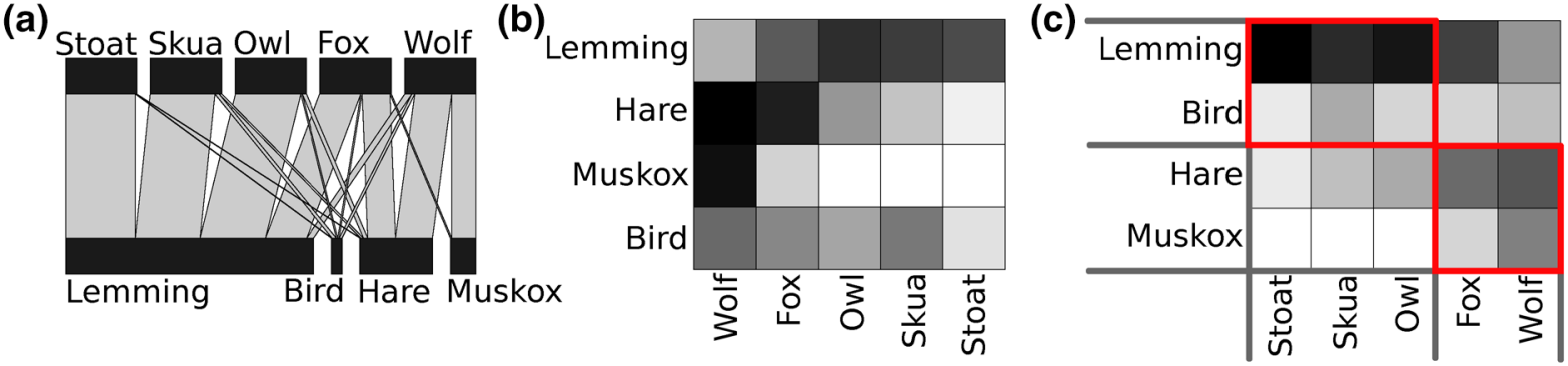
Bipartite network of predator–prey interactions constructed of pooled data from all peninsulas (a), a matrix representation of this network with rows and columns sorted for optimal nestedness, that is, they are packed with increasing row and column totals towards the upper left corner (b), and a matrix representation highlighting identified modular structures in the same network (c). In this latter representation, rows and columns are sorted for optimal modularity. In the bipartite networks, the size of prey categories reflects their total use by all predators, and the width of each link reflects the dietary contribution of a prey category for each specific predator. For both matrix representations, a darker shade of a cell indicates a higher dietary contribution of a prey category for that particular predator. Red boxes indicate identified modules.

**FIGURE 3:**
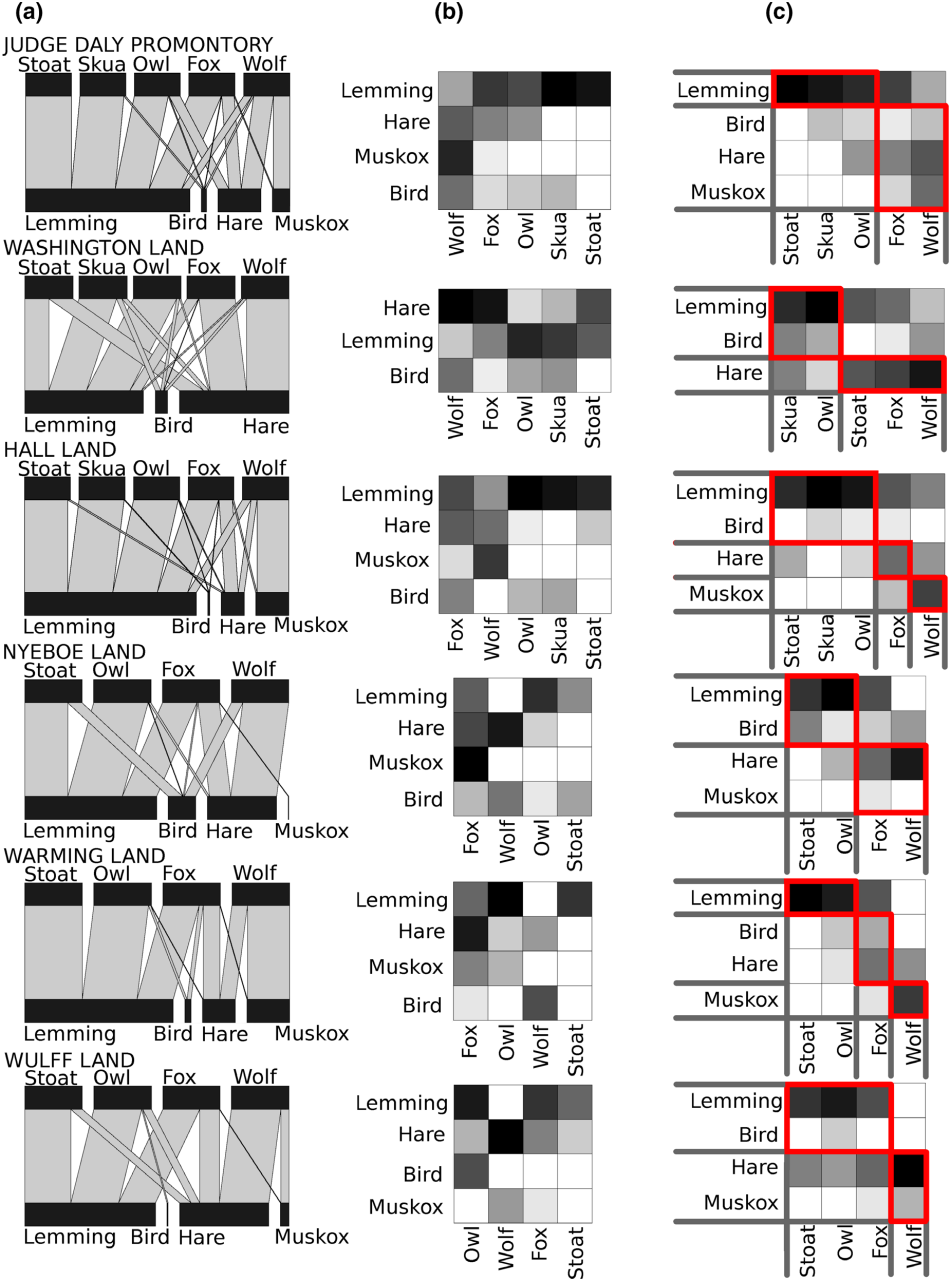
Bipartite networks of predator–prey interactions constructed of data from six geographically separated peninsulas (a), matrix representations of these networks with rows and columns sorted for optimal nestedness, that is, they are packed with increasing row and column totals towards the upper left corner (b), and matrix representations describing modular structures in the same networks (c). In these latter representations, rows and columns are sorted for optimal modularity. For all matrix representations, a darker shade of a cell indicates a higher dietary contribution of a prey category for that particular predator. Red boxes indicate identified modules.

The bipartite networks describing predator–prey interactions were significantly less complex than random expectations, both for the network constructed of data from all sites pooled (Figure 2a) and for the networks constructed of data from each peninsula (Figure 3a). However, there were differences among sites in the degree of complexity (*Z =* 61.60, *p* < .001), where Warming Land and Nyeboe Land were relatively less complex compared to the other sites (Table 2). For the network constructed of data from all sites pooled, 16 of 20 possible predator–prey interactions were observed (Figure 2a), whereas 14 of 20 interactions were observed in the network from Judge Daly Promontory and Hall Land, 14 of 15 interactions were observed in the network from Washington Land, 11 of 16 interactions in the network from Nyeboe Land, and 10 of 16 interactions were observed in the networks from Warming Land and Wulff Land (Figure 3a).

**TABLE 2:** The observed and expected values as well as *Z* scores and associated *p*‐values for weighted connectance, network specialization, specialization asymmetry, predator specialization, prey specialization, nestedness, and modularity, calculated from bipartite predator–prey networks constructed of data from all sites pooled as well of data from each peninsula. Weighted connectance was quantified as the total number of trophic links per species divided by the number of species in the network, network level specialization was quantified using the H2 index, specialization asymmetry was quantified using and index comparing the predator and prey level specializations, the specialization within each trophic level was quantified as the Horn's index of niche overlap, nestedness was quantified using the Weighted‐Interaction Nestedness Estimator (WINE), and modularity was quantified using the Q_w_ index based on the QuanBiMo algorithm.

	Weighted connectance				Network specialization				Specialization asymmetry				Predator level specialization				Prey level specialization				Nestedness				Modularity			
	Obs	Exp	*Z*	*p*	Obs	Exp	*Z*	*p*	Obs	Exp	*Z*	*p*	Obs	Exp	*Z*	*p*	Obs	Exp	*Z*	*p*	Obs	Exp	*Z*	*p*	Obs	Exp	*Z*	*p*
All	0.31	0.49	−72.25	<.001	0.49	0.01	110.58	<.001	−0.33	−0.07	−28.38	<.001	0.34	0.03	27.73	<.001	0.51	0.03	27.73	<.001	0.38	0.22	74.16	<.001	0.29	0.05	4.46	<.001
Judge Daly Promontory	0.32	0.49	−71.24	<.001	0.47	0.01	106.73	<.001	−0.29	−0.07	−26.80	<.001	0.28	0.03	22.64	<.001	0.47	0.03	22.64	<.001	0.84	0.22	71.08	<.001	0.22	0.05	4.72	<.001
Washington Land	0.34	0.5	−74.78	<.001	0.47	0.01	96.46	<.001	−0.34	−0.19	−16.88	<.001	0.39	0.02	38.87	<.001	0.48	0.02	38.87	<.001	0.42	0.27	64.68	<.001	0.30	0.05	3.73	<.001
Hall Land	0.3	0.49	−75.27	<.001	0.68	0.01	149.62	<.001	−0.30	−0.07	−27.00	<.001	0.32	0.03	25.47	<.001	0.64	0.03	25.47	<.001	0.77	0.23	72.61	<.001	0.28	0.05	4.47	<.001
Nyeboe Land	0.27	0.49	−79.97	<.001	0.63	0.01	143.31	<.001	−0.24	0.01	−21.66	<.001	0.47	0.03	32.95	<.001	0.65	0.03	32.95	<.001	0.42	0.25	71.57	<.001	0.29	0.05	3.92	<.001
Warming Land	0.25	0.49	−88.41	<.001	0.89	0.01	209.38	<.001	−0.24	0.01	−21.75	<.001	0.52	0.03	38.45	<.001	0.62	0.03	38.45	<.001	0.56	0.25	70.75	<.001	0.25	0.05	4.05	<.001
Wulff Land	0.29	0.49	−71.00	<.001	0.53	0.01	117.98	<.001	−0.29	<0.01	−26.63	<.001	0.37	0.03	24.95	<.001	0.67	0.03	24.95	<.001	0.62	0.25	72.68	<.001	0.29	0.05	3.72	<.001

### 3.3 Interaction specialization

All networks exhibited more specialized interaction structures than random expectations, with an observed specialization index of 0.49 for all data pooled (Table 2). However, there were differences among the peninsulas in the degree of specialization (*Z =* 51.92, *p* < .001), with Wulff Land (0.89) and Hall Land (0.68), having the highest observed specialization values, while Judge Daly Promontory and Washington Land had the lowest (0.47 for each of these peninsulas).

There was a higher trophic‐level asymmetry in interaction specialization than random expectations, with negative index values for all networks suggesting lower specialization among predators than among prey (Table 2). However, both predators and prey had significantly more specialized interactions than random expectations (Table 2). The networks constructed on data from each peninsula differed in their degree of asymmetry (*Z =* 77.45, *p* < .001) with the networks from Nyeboe Land, Warming Land, and Wulff Land having higher deviations from random expectations in asymmetry than the other sites, while Washington Land had the lowest deviation (Table 2). As with asymmetry in interaction specialization, there were differences among peninsulas in the degree of specialization for predators (*Z =* 66.06, *p* < .001) as well as for prey (*Z =* 53.26, *p* < .001).

All individual predator (Table 3) and prey (Table 4) species had higher species‐level interaction specialization than random expectations. Despite its relatively broad diet, in the network constructed of data from all sites pooled, the wolf had the most specialized interactions among the predators (0.52), followed by the stoat (0.18), and the Arctic fox had the lowest (0.07) (Table 3). For prey, muskoxen had the most specialized interactions with predators (0.53), and birds had the lowest (0.05) (Table 4). However, there were significant differences among the peninsulas in the degree of interaction specialization, for all predators (wolf *Z* = 50.45, *p* < .001; Arctic fox *Z* = 52.11, *p* < .001; stoat *Z* = 48.96, *p* < .001; snowy owl *Z* = 51.43, *p* < .001; skua *Z* = 36.10, *p* < .001) as well as for all prey (muskox *Z* = 43.37, *p* < .001; hare *Z* = 61.68, *p* < .001; lemming *Z* = 59.34, *p* < .001; birds *Z* = 48.11, *p* < .001).

**TABLE 3:** The observed and expected index values, *Z* score, and associated *p*‐value of species level interaction specialization of predators in networks constructed of data from all sites pooled as well from each peninsula. Interaction specialization was quantified using the *d* index, which estimates how strongly a species deviates from a random sampling of the possible interaction partners.

	Wolf				Arctic fox				Stoat				Snowy owl				Skua			
	*d*_obs_	*d*_exp_	*Z*	*p*	*d*_obs_	*d*_exp_	*Z*	*p*	*d*_obs_	*d*_exp_	*Z*	*p*	*d*_obs_	*d*_exp_	*Z*	*p*	*d*_obs_	*d*_exp_	*Z*	*p*
All	0.52	0.01	82.43	<.001	0.07	0.01	9.40	<.001	0.18	0.01	29.59	<.001	0.11	0.01	17.00	<.001	0.13	0.01	19.15	<.001
Judge Daly Promontory	0.42	0.01	66.98	<.001	0.03	0.01	3.83	<.001	0.21	0.01	33.22	<.001	0.05	0.01	7.52	<.001	0.19	0.01	28.13	<.001
Washington Land	0.32	0.01	55.25	<.001	0.11	0.01	21.42	<.001	0.03	0.01	5.11	<.001	0.30	0.01	53.45	<.001	0.18	0.01	35.74	<.001
Hall Land	0.52	0.01	83.48	<.001	0.12	0.01	19.72	<.001	0.11	0.01	18.68	<.001	0.14	0.01	20.12	<.001	0.18	0.01	24.87	<.001
Nyeboe Land	0.63	0.01	91.09	<.001	0.05	0.01	6.67	<.001	0.27	0.01	41.06	<.001	0.27	0.01	37.59	<.001				
Warming Land	0.85	0.01	119.96	<.001	0.16	0.01	22.75	<.001	0.32	0.01	46.75	<.001	0.26	0.01	36.97	<.001				
Wulff Land	0.63	0.01	88.87	<.001	0.02	0.01	1.27	.21	0.10	0.01	13.36	.14	0.14	0.01	19.56	<.001				

**TABLE 4:** The observed and expected index values, *Z* score, and associated *p*‐value of species‐level interaction specialization of prey in networks constructed of data from all sites pooled as well as from each peninsula. Interaction specialization was quantified using the *d* index, which estimates how strongly a species deviates from a random sampling of the possible interaction partners.

	Muskox				Hare				Bird				Lemming			
	*d*_obs_	*d*_exp_	*Z*	*p*	*d*_obs_	*d*_exp_	*Z*	*p*	*d*_obs_	*d*_exp_	*Z*	*p*	*d*_obs_	*d*_exp_	*Z*	*p*
All	0.53	0.01	79.85	<.001	0.35	0.01	58.03	<.001	0.05	0.01	6.47	<.001	0.41	0.01	61.26	<.001
Judge Daly Promontory	0.54	0.01	85.98	<.001	0.32	0.01	52.41	<.001	0.09	0.01	13.06	<.001	0.32	0.01	49.47	<.001
Washington Land					0.43	0.01	78.30	<.001	0.14	0.01	23.93	<.001	0.37	0.01	65.15	<.001
Hall Land	0.74	0.01	121.20	<.001	0.24	0.01	37.39	<.001	0.08	0.01	10.11	<.001	0.36	0.01	57.86	<.001
Nyeboe Land	0.00	0.01	−1.24	<.001	0.53	0.01	77.27	<.001	0.22	0.01	30.75	<.001	0.55	0.01	80.59	<.001
Warming Land	0.78	0.01	106.90	<.001	0.32	0.01	44.95	<.001	0.20	0.01	28.06	<.001	0.70	0.01	103.42	<.001
Wulff Land	0.35	0.01	47.83	<.001	0.25	0.01	34.92	<.001	0.15	0.01	20.04	<.001	0.52	0.01	73.31	<.001

### 3.4 Network nestedness and modularity

All networks were significantly more nested than random expectations (Table 2), with intermediate to high levels of nestedness both for the network constructed of data from all peninsulas pooled (Figure 2b) and for the networks constructed from each peninsula separately (Figure 3b). However, there were differences among the peninsulas in the degree of nestedness (*Z =* 77.45 and *p* < .001), where Judge Daly Promontory (0.84) and Hall Land (0.77) had the highest nestedness values, and Washington Land and Nyeboe Land had the lowest (0.42 for each site) (Table 2).

Both the network constructed of data from all peninsulas pooled (Figure 2c) and of data from each peninsula (Figure 3c) were significantly more modular than random expectations, but all networks showed only intermediate levels of modularity (Table 2). As with all other network metrics, the degree of modularity differed among the sites (*Z* = 77.45 and *p* < .001), with Washington Land having the most and Judge Daly Promontory having the least modular networks (Table 2). Two modules were identified in the network containing data from all peninsulas: one with stoats, skuas, and owls as predators and lemmings and birds as prey; and another module with foxes and wolves as predators and hare and muskox as prey (Figure 2c). Two modules were also identified in the network from Judge Daly Promontory, Washington Land, Nyeboe Land and Wulff Land, although the modules did not contain the same predators and prey on the different peninsulas (Figure 3c). Three modules were identified in the networks on Hall Land and Warming Land. For these networks, the predators were relatively consistent, with wolves and Arctic foxes forming their own modules and stoats and snowy owls forming a third module, with the addition of skuas on Hall Land. However, the composition of prey modules differed between these two networks, with birds forming a module with lemmings on Hall Land, while birds formed a module with hares on Warming Land.

## 4 DISCUSSION

Our results support the hypotheses that these predator–prey networks would show limited complexity and nested interaction structures. However, we also found relatively high specialization of the predator–prey interactions and significant but modest modular structures. Furthermore, we observed spatial variation in predator diet composition among the different sample sites, resulting in spatial variations in the structures of the predator–prey networks. Considering that this study was done in one of the northernmost land areas on Earth, we suggest that the low productivity associated with these high latitudes has shaped the interaction structures between predators and prey. Our study further highlights the importance of local factors in shaping food web structures by changing the number of species interactions. Such modulations would support recent suggestions of the central importance of context dependencies for biological processes (Catford et al., 2022) and highlight that ecosystems are shaped by processes acting simultaneously across different spatial scales (Kolasa & Pickett, 1991). We suggest that the low productivity associated with the high latitude may have set some limits on the complexity and structure of the trophic interactions, but that regional variation in environmental characteristics caused variation in the realized interaction structures within these limits. Such dependencies of processes across different spatial scales may, to some extent, explain the often‐complex ecological responses to large‐scale environmental perturbations, such as climate change (Walther et al., 2002), and highlight the difficulties in managing environmental resources in variable environments (Pickett et al., 1997).

We found that the predator–prey networks contained less complex and more specialized structures than random expectations. We note that these random expectations were generated from null models in which predators on average utilized an equal proportion of all prey categories available. The low level of complexity is consistent with our hypothesis based on the low productivity in this high arctic region. We suggest that the low network complexity was caused by a high interaction specialization, in which each prey species experienced predation from a relatively unique set of predators (Blüthgen et al., 2008). Since the degree of ecological specialization is thought to increase with high diversity (Araújo et al., 2011) and high complexity (Guimaraes, 2020), it is unclear what has caused the high level of interactions specialization in this relatively simple ecosystem. However, we suggest that the higher level of interaction specialization of prey than of predators may have been caused by constraints in prey sizes for small predators coupled with opportunistic feeding habits by larger ones. This would result in a pattern where each prey would be utilized by a distinct set of predators, whereas there would be more overlap among predators in the use of prey. We encourage further studies evaluating the influence of resource utilization strategies on network complexity.

While previous studies have shown that predator–prey interactions primarily form modular structures (Bascompte et al., 2003), we hypothesized that the low productivity and subsequent energetic constraints imposed on predators would result in nested structures of the trophic interactions among predators and prey. Our results partly supported this hypothesis, as we observed only modest modularity but more pronounced nested structures. Moreover, we observed substantial spatial variation in the observed nestedness as well as in the identified modules, both for predators and prey. This reiterates the importance of regional and local environmental conditions for trophic network structures. Since both nested and modular network structures are important forces for ecosystem stability, resilience, and productivity (Bascompte et al., 2003; Miranda et al., 2013; Van der Putten et al., 2004), our results highlight the interactive effects of species traits and environmental conditions for shaping ecosystem properties (e.g., Dalerum et al., 2012).

The high‐arctic ecosystem of Ellesmere Island and north Greenland has a simple predator–prey community with most predators preying on lemmings, similar to the ecosystem on east Greenland (Gilg et al., 2006). Our observations suggest that mammals were the most important prey item for all predators, and lemmings were the most important prey for all predators except for wolves. Lemmings have previously been highlighted as a keystone species in arctic ecosystems (Dalerum & Angerbjörn, 2000) and are important to the breeding success and population dynamics of most arctic predators (Dudenhoeffer et al., 2021; Elmhagen et al., 2000). Lemmings are sensitive to climate change, and a decline in lemming density can have a profound impact on predators (Ehrich et al., 2020), including a huge impact on both stoat and snowy owl reproductive performance and population size (Schmidt et al., 2012). Arctic fox, stoat, snowy owl, and skua are lemming specialists but use alternative prey during the low density of lemming populations (Gilg et al., 2006) or expand their geographic range (Schmidt et al., 2012). In our case, the main alternative prey was hare. Our observation also supported previous observations of the importance of both muskox and hare as prey for wolves (Dalerum et al., 2018; Marquard‐Petersen, 1998; Mech, 1988) and highlighted that hares may function as an important alternative prey to lemmings for smaller predators and to muskoxen for wolves.

We do recognize some limitations and caveats with our study. First, the sample sizes varied greatly among predators as well as among sites. Therefore, the precision of the dietary estimates was uncertain at some sites and for some predator species. Henrik's Ø was also excluded from the network analysis since we did not have sufficient data from this site. However, the data pooled from all sites likely provided a sufficient estimation of dietary determinations with very high accuracy, whereas individual sites showed a relatively high margin of error. We note that samples from skuas were collected on 3 sites, and our study did not include any data on gyrfalcons, common ravens, or glaucus gulls. The lack of samples from gyrfalcon was most likely due to very low abundance in the study region (Potapov & Sale, 2005), whereas neither ravens nor glaucus gulls produce easily identified regurgitation pellets for diet quantification. Second, there was a relatively low resolution in the taxonomic identification of avian prey remains. This was caused by a high frequency of damaged feather structures, which resulted in approximately half of the avian prey remains being identified as unknown birds. Therefore, birds were pooled into one dietary category for the network analysis. However, since the avian prey category had relatively limited dietary contributions, we do not believe that this grouping had strong consequences on the identified network structures. Finally, we used samples with an unknown temporal resolution. Both lemmings (Braestrup, 1941) and hares (Dalerum et al., 2017) appear to undergo temporal fluctuations in abundance in this region. Since feces and regurgitation pellets likely last several years in the high Arctic, it is important to point out that the observed network structures likely reflect dietary contributions averaged across several demographic phases of individual prey species. Therefore, temporally resolved networks would be highly informative but would require repeated collection of fresh material within a single year.

To conclude, we found limited complexity in the observed predator–prey networks, which is consistent with our hypothesis that low primary productivity would limit trophic complexity in this high arctic environment. However, our results only partially supported our hypothesis of nested rather than modular interaction structures in these predator–prey networks, since we found both nested and modular structures in all networks. Contrary to our hypothesis of limited specialization, we found relatively high levels of specialization among both predators and prey. All network properties differed among the peninsulas, which suggests that partial variation in relative prey abundance or environmental factors may modulate the trophic interaction structures within some limits dictated by primary productivity. We suggest that such scale dependencies may explain the often‐complex ecological responses to environmental perturbations and highlight the difficulties in managing environmental resources under environmental change. Understanding the regulation of trophic network structures at different spatial scales may be crucial for effective ecosystem management in the face of environmental change and uncertainty.

## AUTHOR CONTRIBUTIONS

**Muzit Abrham:** Formal analysis (supporting); investigation (equal); methodology (supporting); writing – original draft (equal). **Karin Norén:** Investigation (supporting); project administration (supporting); resources (supporting); supervision (supporting); writing – review and editing (supporting). **Jordi Bartolomé Filella:** Funding acquisition (supporting); investigation (supporting); project administration (supporting); writing – review and editing (supporting). **Anders Angerbjörn:** Investigation (supporting); project administration (supporting); resources (supporting); writing – review and editing (supporting). **Nicolas Lecomte:** Investigation (supporting); writing – review and editing (supporting). **Patrícia Pečnerová:** Investigation (supporting); writing – review and editing (supporting). **Susana Freire:** Investigation (supporting); writing – review and editing (supporting). **Fredrik Dalerum:** Conceptualization (lead); data curation (lead); formal analysis (lead); funding acquisition (lead); investigation (equal); methodology (lead); project administration (lead); resources (lead); supervision (lead); writing – original draft (equal).

## FUNDING INFORMATION

Field data collection was logistically supported by the Swedish Polar Research Secretariat by offering participation in the expeditions Petermann2015 and Ryder2019. Financial support was further provided by the Spanish Ministry of Economy and Competitiveness (RYC‐2013‐14662), by the Spanish Ministry of Science and Innovation (PID2019‐107862RB‐I00 and PID2022‐137336OB‐I00), the Spanish National Research Council (LINKA20417) and by the Canada Research Chair program.

## CONFLICT OF INTEREST STATEMENT

The authors declare no conflict of interests.

## Supporting information


Data S1.

## Data Availability

Data for this paper are available in the Table S1.
