# Supplementary material for: Properties of vertebrate predator–prey networks in the high Arctic
Source: Ecol Evol. 2024 May 30;14(6):e11470. doi: 10.1002/ece3.11470 (PMC11137350; doi:10.1002/ece3.11470)
Supplement: Supplementary file 1 — Data S1. [file ECE3-14-e11470-s001.pdf]

**Supplementary Material**

# Properties of vertebrate predator-prey in the high Arctic

Muzit Abrham[1], Karin Norén[1], Jordi Bartolomé Filella[2], Anders Angerbjörn[1], Nicolas Lecomte[3], Patrícia Pečnerová[4], Susana Freire[5], Fredrik Dalerum[1,5,6*]

1. Department of Zoology, Stockholm University, 106 91 Stockholm, Sweden
2. Department of Animal and Food Science, Autonomous University of Barcelona, Barcelona, Cataloña, Spain
3. Department of Biology, University of Moncton, Canada
4. Department of Biology, University of Copenhagen, Denmark
5. Biodiversity Research Institute (CSIC-Univ. Oviedo Princ. Asturias), Research Building, Mieres Campus, 33600 Mieres, Asturias, Spain
6. Mammal Research Institute, Department of Zoology and Entomology, University of Pretoria, Pretoria, South Africa

* Corresponding author at: Biodiversity Research Institute (CSIC-Univ. Oviedo Princ. Asturias), Research Building, Mieres Campus, 33600 Mieres, Asturias, Spain. E-mail: fredrik.dalerum@csic.es

**Table S1.** Estimated contributions of all prey classes to the diet of wolf, arctic fox, stoat, snowy owl, and skua at each site.

| **WOLF** | Judge Daly Promontory (*N*=16) | Washington Land (*N*=26) | Hall Land (*N*=25) | Nyeboe Land (*N*=5) | Warming Land (*N*=12) | Henrik's Ø (*N*=1) | Wulff Land (*N*=7) |
|---|---|---|---|---|---|---|---|
| **Mammal** | 97% | 91% | 100% | 80% | 69% | 100% | 100% |
| Muskox | 31% | 0% | 66% | 0% | 44% | 100% | 14% |
| Arctic fox | 6% | 0% | 0% | 0% | 0% | 0% | 0% |
| Hare | 38% | 87% | 12% | 80% | 17% | 0% | 86% |
| Lemming | 16% | 4% | 18% | 0% | 0% | 0% | 0% |
| Unk.mammal | 6% | 0% | 4% | 0% | 8% | 0% | 0% |
| **Bird** | 3% | 8% | 0% | 20% | 0% | 0% | 0% |
| Anseriformes | 0% | 1% | 0% | 0% | 0% | 0% | 0% |
| Galliformes | 0% | 7% | 0% | 0% | 0% | 0% | 0% |
| Charadriiformes | 3% | 0% | 0% | 0% | 0% | 0% | 0% |
| Passeriformes | 0% | 0% | 0% | 0% | 0% | 0% | 0% |
| Unk.bird | 0% | 0% | 0% | 20% | 0% | 0% | 0% |
| **Plants** | 0% | 0% | 0% | 0% | 0% | 0% | 0% |
| **Arthropods** | 0% | 0% | 0% | 0% | 0% | 0% | 0% |
| **Miscellaneous** | 0% | 1% | 0% | 0% | 31% | 0% | 0% |

| **ARCTIC FOX** | Judge Daly Promontory (*N*=100) | Washington Land (*N* =130) | Hall Land (*N*=176) | Nyeboe Land (*N*=84) | Warming Land (*N*=53) | Henrik's Ø (*N*=22) | Wulff Land (*N*=92) |
|---|---|---|---|---|---|---|---|
| **Mammal** | 94% | 96% | 94% | 93% | 89% | 90% | 97% |
| Muskox | 2% | 0% | 3% | 1% | 1% | 0% | <2% |
| Arctic fix | 0% | 0% | 0% | 0% | 0% | 0% | 0% |
| Hare | 25% | 73% | 28% | 35% | 28% | 19% | 33% |
| Lemming | 67% | 23% | 63% | 57% | 61% | 71% | 62% |
| Unk.mammal | 0% | 0% | 0% | 0% | 0% | 0% | 0% |
| **Bird** | 1% | 1% | 1% | 2% | 7% | 1% | 0% |
| Anseriformes | <1% | 1% | 0% | 1% | 3% | 0% | 0% |
| Galliformes | 0% | 0% | 1% | 1% | 2% | <1% | 0% |
| Charadriiformes | 0% | 0% | 0% | 0% | 0% | 0% | 0% |
| Passeriformes | 0% | 0% | 0% | 0% | 1% | 0% | 0% |
| Unk.bird | 0% | 0% | 0% | 0% | 0% | 1% | 0% |
| **Plants** | 4% | 3% | 5% | 5% | 3% | 9% | 1% |
| **Arthropods** | 0% | 0% | 0% | 0% | 1% | 0% | <1% |
| **Miscellaneous** | 1% | 0% | 0% | 0% | 0% | 0% | 1% |

| **STOAT** | Judge Daly Promontory (*N*=227) | Washington Land (*N*=2) | Hall Land (*N*=18) | Nyeboe Land (*N*=4) | Warming Land (*N*=4) | Henrik's Ø (*N*=2) | Wulff Land (*N*=5) |
|---|---|---|---|---|---|---|---|
| **Mammal** | 100% | 100% | 100% | 74% | 100% | 50% | 98% |
| Muskox | 0% | 0% | 0% | 0% | 0% | 0% | 0% |
| Arctic fox | 0% | 0% | 0% | 0% | 0% | 0% | 0% |
| Hare | 0% | 50% | 6% | 0% | 0% | 0% | 20% |
| Lemming | 100% | 50% | 94% | 74% | 100% | 50% | 78% |
| Unk.mammal | 0% | 0% | 0% | 0% | 0% | 0% | 0% |
| **Bird** | 0% | 0% | 0% | 25% | 0% | 50% | 0% |
| Anseriformes | 0% | 0% | 0% | 0% | 0% | 0% | 0% |

| | | | | | | | |
|---|---|---|---|---|---|---|---|
| Galliformes | 0% | 0% | 0% | 0% | 0% | 0% | 0% |
| Charadriiformes | 0% | 0% | 0% | 0% | 0% | 0% | 0% |
| Passeriformes | 0% | 0% | 0% | 25% | 0% | 50% | 0% |
| Unk.bird | 0% | 0% | 0% | 0% | 0% | 0% | 0% |
| **Plants** | 0% | 0% | 0% | 0% | 0% | 0% | 0% |
| **Arthropods** | 0% | 0% | 0% | 0% | 0% | 0% | 0% |
| **Miscellaneous** | 0% | 0% | 0% | 1% | 0% | 0% | 2% |

| **SNOWY OWL** | Judge Daly Promontory (*N*=25) | Washington Land (*N*=32) | Hall Land (*N*=135) | Nyeboe Land (*N*=50) | Warming Land (*N*=61) | Henrik's Ø (*N*=16) | Wulff Land (*N*=67) |
|---|---|---|---|---|---|---|---|
| **Mammal** | 98% | 94% | 99% | 99% | 93% | 94% | 98% |
| Muskox | 0% | 0% | 0% | 0% | 0% | 0% | 0% |
| Arctic fox | 0% | 0% | 0% | 0% | 0% | 0% | 0% |
| Hare | 24% | 3% | 2% | 4% | 1% | 0% | 15% |
| Lemming | 74% | 91% | 97% | 95% | 92% | 94% | 83% |
| Unk.mammal | 0% | 0% | 0% | 0% | 0% | 0% | 0% |
| **Bird** | 2% | 6% | 1% | 1% | 5% | 6% | 2% |
| Anseriformes | 0% | 0% | 0% | 0% | 0% | 0% | 0% |
| Galliformes | 0% | 1% | <1% | 0% | 0% | 6% | 2% |
| Charadriiformes | 0% | 0% | 0% | 1% | 2% | 0% | 0% |
| Passeriformes | 0% | 0% | 0% | 0% | 1% | 0% | 0% |
| Unk.bird | 2% | 6% | 1% | 0% | 2% | 0% | 0% |
| **Plants** | 0% | 0% | 0% | 0% | 0% | 0% | 0% |
| **Arthropods** | 0% | 0% | 0% | 0% | 0% | 0% | 0% |
| **Miscellaneous** | 0% | 0% | 0% | 0% | 2% | 0% | 0% |

| **SKUA** | Judge Daly Promontory (*N*=15) | Washington Land (*N*=9) | Hall Land (*N*=6) | Nyeboe Land (*N*=0) | Warming Land (*N*=0) | Henrik's Ø (*N*=0) | Wulff Land (*N*=0) |
|---|---|---|---|---|---|---|---|
| **Mammal** | 97% | 89% | 98% | | | | |
| Muskox | 0% | 0% | 0% | | | | |
| Arctic fox | 0% | 0% | 0% | | | | |
| Hare | 0% | 11% | 0% | | | | |
| Lemming | 84% | 78% | 98% | | | | |
| Unk.mammal | 13% | 0% | 0% | | | | |
| **Bird** | 3% | 11% | 2% | | | | |
| Anseriformes | 0% | 0% | 0% | | | | |
| Galliformes | 0% | 1% | 0% | | | | |
| Charadriiformes | 0% | 0% | 0% | | | | |
| Passeriformes | 0% | 0% | 0% | | | | |
| Unk.bird | 3% | 10% | 2% | | | | |
| **Plants** | 0% | 0% | 0% | | | | |
| **Arthropods** | 0% | 0% | 0% | | | | |
| **Miscellaneous** | 0% | 0% | 0% | | | | |

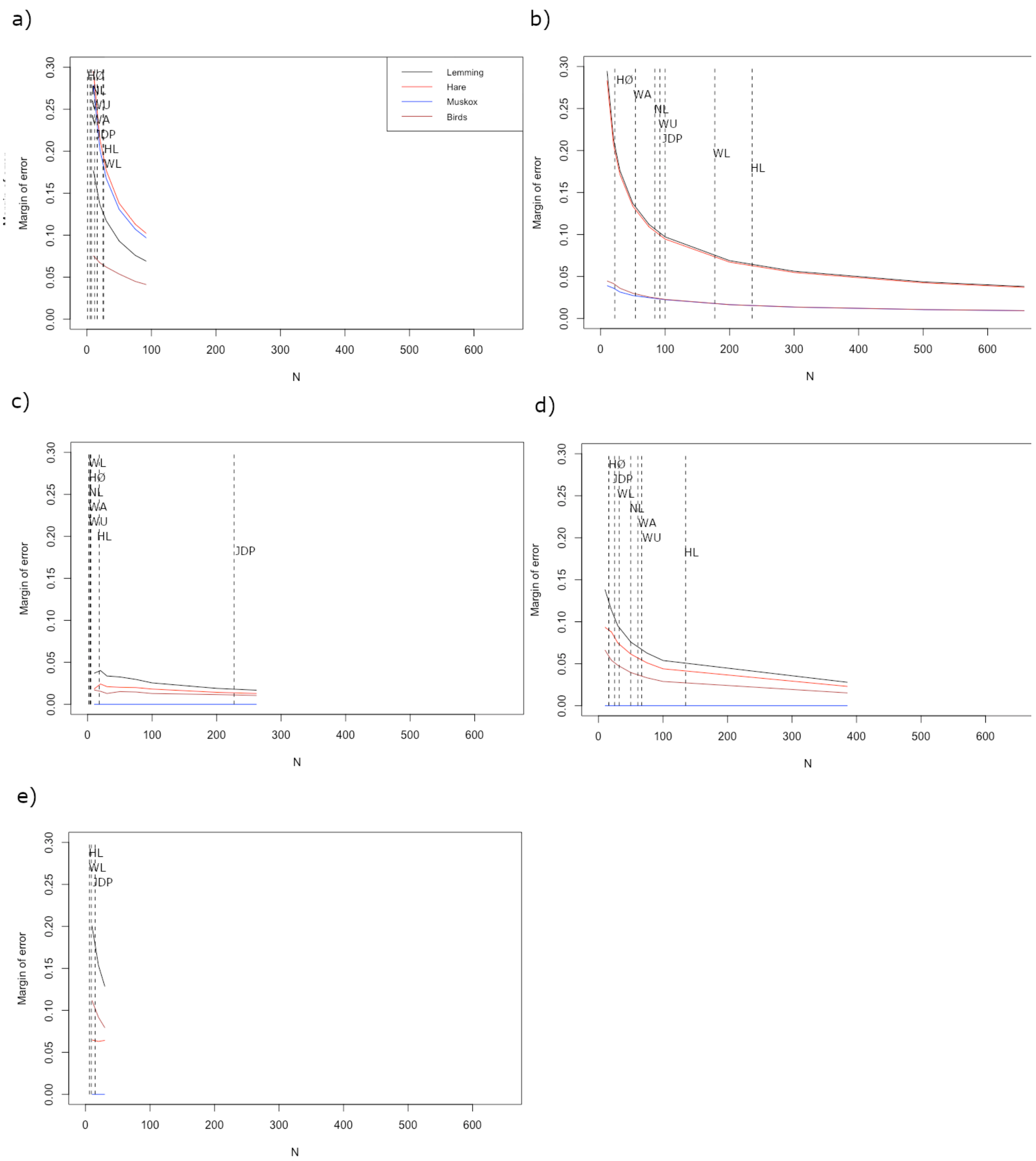


**Figure S1.** The effect of sample size on the precision of estimation of dietary contribution, quantified as the margin of error, of each prey class to the diet of a) wolf, b) arctic fox, c) stoat, d) snowy owl, and e) skua. Each graph ends at the full sample size for respective species, and the vertical lines represents the sample sizes at the different sample sites; JDP - Judge Daly Promontory, WL - Washington Land, HL - Hall land, NL - Nyeboe Land, WA - Warming Land, HØ- Henrik's Ø, and WU - Wulff Land.
